# Using Geospatial Data and Random Forest To Predict PFAS Contamination in Fish Tissue in the Columbia River Basin, United States

**DOI:** 10.1021/acs.est.3c03670

**Published:** 2023-09-05

**Authors:** Nicole M. DeLuca, Ashley Mullikin, Peter Brumm, Ana G. Rappold, Elaine Cohen Hubal

**Affiliations:** † Center for Public Health and Environmental Assessment, Office of Research and Development, U.S. Environmental Protection Agency, Research Triangle Park, North Carolina 27709, United States; ‡ Region 08, Water Division, U.S. Environmental Protection Agency, Helena, Montana 59626, United States

**Keywords:** variable importance, industry, land cover, sources, Washington, Oregon, tribes

## Abstract

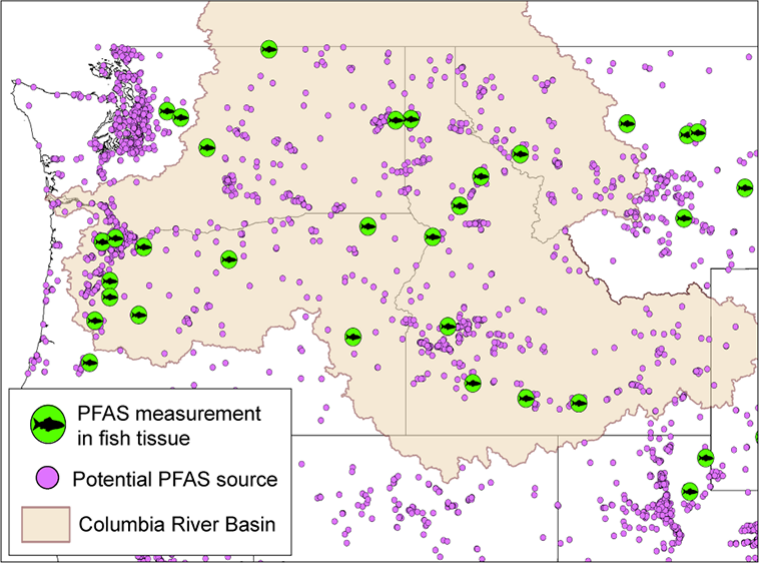

Decision makers in the Columbia River Basin (CRB) are currently challenged with identifying and characterizing the extent of per- and polyfluoroalkyl substances (PFAS) contamination and human exposure to PFAS. This work aims to develop and pilot a methodology to help decision makers target and prioritize sampling investigations and identify contaminated natural resources. Here we use random forest models to predict ∑PFAS in fish tissue; understanding PFAS levels in fish is particularly important in the CRB because fish can be a major component of tribal and indigenous people diet. Geospatial data, including land cover and distances to known or potential PFAS sources and industries, were leveraged as predictors for modeling. Models were developed and evaluated for Washington state and Oregon using limited available empirical data. Mapped predictions show several areas where detectable concentrations of PFAS in fish tissue are predicted to occur, but prior sampling has not yet confirmed. Variable importance is analyzed to identify potentially important sources of PFAS in fish in this region. The cost-effective methodologies demonstrated here can help address sparsity of existing PFAS occurrence data in environmental media in this and other regions while also giving insights into potentially important drivers and sources of PFAS in fish.

## Abstract 2

High-exposure risk populations and data-limited regions can use geospatial data and random forest models to efficiently identify potential hotspots of PFAS in environmental media like fish to target their sampling and remediation efforts.

## 1 Introduction

Per- and polyfluoroalkyl substances (PFAS) are man-made, pervasive compounds that are widely used in a range of industrial processes and consumer products.^1^ Currently in the United States, it is estimated that millions of homes receive PFAS-contaminated drinking water, and local testing indicates widespread contamination of environmental media.^2,3^ Human exposure to PFAS is thought to mainly occur through dietary and drinking water intake.^4^ With PFAS exposure being a growing concern for governments, communities impacted by contamination, as well as the general public, models and tools that use available spatial data to identify hotspots and important predictors of PFAS contamination in environmental media are actively being developed.^5−9^ Previous studies have developed predictive models to identify PFAS contamination in groundwater and drinking water, often in smaller regions that are relatively rich with PFAS occurrence data. These groundwater and drinking water models generally include expected sources of high levels of PFAS contamination from aqueous film-forming form (AFFF) use such as that at airports, fire training facilities, and military installations.^6−9^ However, few studies evaluate the potential impact of other types of PFAS-related sources and industries or account for potential contamination from PFAS other than perfluorooctanesulfonate (PFOS) and perfluorooctanoate (PFOA).^6,8^ Additionally, few if any studies have developed these predictive models for other environmental media such as fish tissue.

The Columbia River Basin (CRB) is home to high fish-consuming populations, such as tribal fish consumers and subsistence fishers.^10^ Tribal people in the CRB have relied on native fish species for physical, cultural, and spiritual sustenance for thousands of years.^11^ However, increasing population and human activity in this region over the past few decades poses a growing risk of impaired water quality and chemical contaminants in locally caught fish.^10,12^ A survey conducted by USEPA from 1989 to 1994 found that members of tribal nations in the CRB ate up to 11 times more fish than the general U.S. population, indicating that they could be a particularly vulnerable population to chemical exposures through their diet.^13^ Several years after this survey, USEPA collected samples of fish frequently eaten by tribal nations in the CRB and found metals, pesticides, and/or organic chemical pollutants in all species.^14^ Other studies have found significant levels of toxic chemical pollutants in fish and surface waters in the CRB, prompting fish consumption advisories to warn the public about potential health risks of eating certain fish in particular locations.^10,15−18^ USEPA, in conjunction with tribal governments, states, and localities, formed the Columbia River Toxics Reduction Working Group (now called the Columbia River Basin Restoration Program Working Group) in 2005 to understand and reduce toxic chemicals in the basin.^10,11^ However, the amount and frequency of sampling in the CRB has decreased since the 1990s and increased monitoring, coordination, and exchange of information across the basin has been recommended.^13,19^

**Figure 1:**
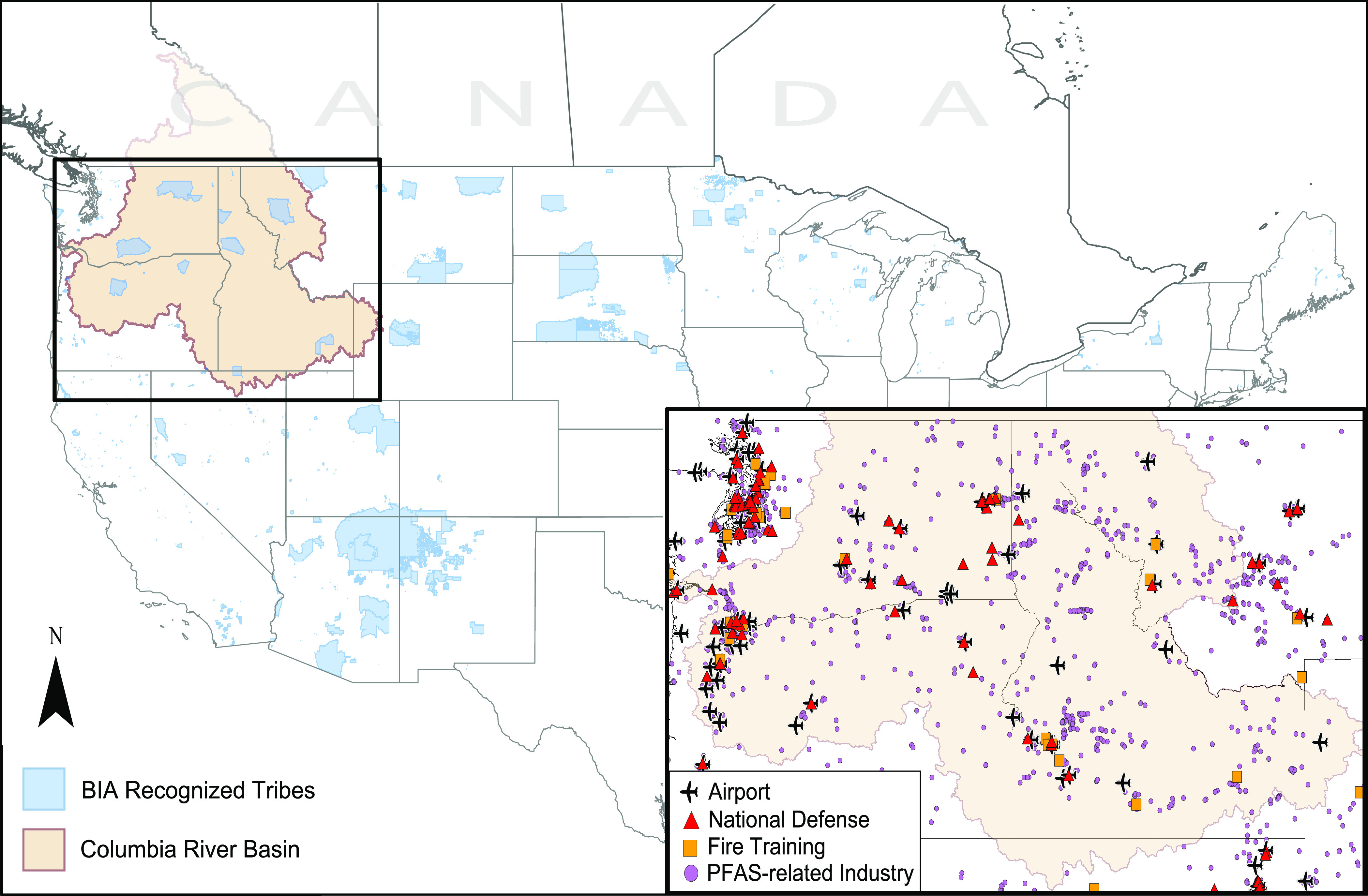
Map of the contiguous United States and southern Canada showing the extent of the Columbia River Basin (shaded in beige) and its incorporated states (text). Bureau of Indian Affairs (BIA) recognized tribes are shaded in blue. Inset map shows the Basin and potential PFAS sources including national defense sites (red triangle), fire training facilities (orange square), airports (airplane symbol), and PFAS-related industry facilities from USEPA’s ECHO database (purple circles).

While PFAS contamination can be presumed near well-studied sources,^5^ the sheer number of those and additional potential PFAS sources throughout a larger region of interest, as well as unknowns regarding specific facilities’ PFAS use, lack of ground truthing data, and uncertain fate and transport properties of PFAS in various environment media, can make sampling prioritization for resource-limited entities overwhelming and complex (Figure 1). To address the sparsity of existing PFAS occurrence data in this region, an efficient methodology is needed to design cost-effective sampling campaigns. This study leverages existing PFAS occurrence data in fish tissue, publicly available geospatial data, and random forest modeling to identify locations with potential PFAS contamination in fish and important sources in the Columbia River Basin. We pilot a broadly applicable modeling workflow that can help decision-makers in the region target and prioritize their sampling investigations and efficiently identify contaminated natural resources.

## 2 Materials and Methods

### 2.1 Study Area

The Columbia River Basin is a large watershed located in the northwestern United States and southwestern Canada that drains an area of about 666,700 square kilometers.^20^ The basin spans across 7 U.S. states (Washington, Oregon, Idaho, Montana, Wyoming, Nevada, and Utah), 16 federally recognized tribal reservations, and extends northward into British Columbia, Canada (Figure 1). The Columbia River is the fourth largest river in North America, beginning in the Canadian Rocky Mountains and emptying into the Pacific Ocean in Washington and Oregon.^21^ Major tributaries in the basin that feed into the Columbia River include the Snake River, Kootenai River, Clark Fork-Pend Oreille River, Willamette River, and Yakima River.^20^ Geography and land cover varies vastly throughout the Basin including areas dominated by rainforests, mountains, deserts, and dry plateaus.^22^ This study focuses on the states of Washington (WA) and Oregon (OR), where the most abundant fish tissue occurrence data in the CRB were identified. The study aims to develop and pilot a workflow from these two states that can later be applied to the rest of the CRB and beyond.

### 2.2 Fish Tissue Occurrence Data

In Washington and Oregon, measurements of PFAS in fish tissue were downloaded from USEPA’s PFAS Analytic Tools (PAT), a data analytic hub for PFAS data measured in various environmental media.^23^ The fish tissue measurement data acquired from the PAT hub was pulled from USEPA’s Water Quality Portal,^24^ where states, tribes, and other organizations can upload their water quality data directly into a central database, and from USEPA’s National Rivers and Streams Assessment.^25^ In addition to the data collected from PAT, other fish tissue measurement data were acquired from by Washington Department of Ecology’s Environmental Information Management (EIM) System.^26^

The fish tissue samples in the dataset obtained from the above sources were collected over years 2008 through 2019. Data from any fish species were included in this study due to the limited availability of data in this region. The recorded fish species included brook trout/sea trout, brown bullhead, channel catfish, common carp, cutthroat trout, largemouth bass, largescale sucker, mountain whitefish, northern pikeminnow, peamouth, pumpkinseed, rainbow trout, redband trout, steelhead trout, smallmouth bass, tench, tyee sucker, walleye, and yellow perch. While many of these fish species are nonmigratory or locally migratory, others such as trout, channel catfish, and walleye have been observed migrating expansive distances for spawning.^27^ The lifespans of these fish are typically around 10 years or less on average except for common carp, bass, tench, and walleye which can live for longer periods of time.^27^ Most of these fish species are lower trophic levels being herbivores, invertivores, and/or piscivores; however, bass can also be higher trophic levels and have partially carnivorous diets.^27^

The data were filtered to only include fillets with skin on in order to obtain better correspondence between the different datasets for the analyses, which produced PFAS measurement data for 45 samples. Several of these fish samples were collected in similar locations or water bodies, but during different years and sampling campaigns. Measurements from different fish specimens, including those of varying species, that were sampled at the same location on the same day were averaged to a single fish sample, given the limited availability and spatial resolution of this data.

### 2.3 Geospatial Data

Locational data have previously been suggested as a starting point for identifying potential PFAS exposure hotspots.^5^ Geospatial data used in this study was acquired from USEPA’s PAT data hub,^23^ which provides downloadable location data about industries, military, and aviation facilities that have been registered through USEPA’s Enforcement and Compliance History Online (ECHO) database and have some relevance to PFAS use or potential discharge.^28^ ECHO industries include sites used for fire training, aviation, national defense, mining and refining, landfills, metal coating, metal machinery manufacturing, industrial gas, glass products, furniture and carpeting, electronics, consumer products, cleaning product manufacturing, chemical manufacturing, cement manufacturing, petroleum, industrial gas, paints and coatings, oil and gas, plastics and resins, printing, paper mills, and textiles. Other than for most military installations, this dataset does not include any actual emissions data or confirmation about each facility’s PFAS use or discharge, but these industry points are treated as potential sources in this study. Wastewater treatment plant locations were downloaded from USEPA’s Integrated Compliance Information System National Pollutant Discharge Elimination System (ICIS-NPDES).^29^ Land cover data were downloaded from the U.S. Geological Survey’s National Land Cover Database.^30^

In order to create spatial units relevant to fish populations where predictions would be made, major rivers, streams, and lakes where fishing was likely to occur were identified using past or current fish consumption advisory lists and locational information from previous sampling in the existing fish tissue PFAS dataset.^31−33^ In ArcMap, points were added along all of these identified waterbodies at 15 km distance apart from each other. Where two or more waterbodies intersect or are near each other, points along one waterbody may be closer than 15 km to points along other waterbodies. PFAS-related industry location data were quantified by calculating the geodesic distance from each waterbody point to the nearest facility for each industry type.^6^ Existing fish tissue PFAS occurrence data were also matched to the nearest waterbody point. A 5 km buffer was drawn around each waterbody point within which the percent of land cover classified as natural land (e.g., forests), agricultural land (e.g., crop fields), or developed land (e.g., urban impervious surfaces) was calculated in order to represent a snapshot of each point’s local environment type. All geospatial calculations were done in ArcMap Desktop version 10.8.1.^34^ Correlations between the quantified spatial data variables were calculated using the Pearson method.^35^

### 2.4 Statistical Methodology

The PFAS reported in the collated fish tissue occurrence dataset varied by sampling campaign; therefore, only common chemicals between all samples were used for analysis. These included perfluorobutane sulfonate (PFBS), perfluorodecanoic acid (PFDA), perfluorododecanoic acid (PFDoA), perfluoroheptanoic acid (PFHpA), perfluorohexane sulfonate (PFHxS), perfluorohexanoic acid (PFHxA), perfluorononanoic acid (PFNA), perfluoroundecanoic acid (PFUnA), PFOS, and PFOA. A ∑PFAS (ng/g) concentration was calculated by summing all detected PFAS concentrations for each fish tissue sample to represent any PFAS exposure that may have been acquired through consumption of the fish. Because the limits of detection were not reported for all sampling campaigns or for all chemicals measured and because the combined dataset was not strongly zero-inflated, nondetects within the dataset were treated as zeros. Summary statistics were calculated for each PFAS concordant between datasets and for the ∑PFAS metric. Modeling to predict ∑PFAS in fish tissue at each waterbody point including unmonitored locations was performed using a random forest model, which has been used in prior PFAS modeling applications.^8^ Random forest models consist of an ensemble of decision trees run in parallel on random subsets of the data and can predict either continuous (regression) or categorical (classification) outputs.^36,37^ The number of trees and number of variables used at each node in the trees (mtry) can be specified by the user. Predictions from the ensemble of decision trees are determined by calculating the average value for regression models or the majority vote for classification models.

Quantified spatial variables at waterbody points that were matched to fish tissue occurrence data were used to develop and then evaluate random forest regression models (*n* = 45). The calculated ∑PFAS metric was used as a continuous response variable in the models. Using a 100-iteration Monte Carlo holdout scheme to evaluate the models, the matched waterbody and fish tissue occurrence dataset was randomly split 100 times into 80% of the data used to train the models and 20% of the data used for holdout cross-validation. Reported model evaluation metrics—mean absolute error (MAE), root mean square error (RMSE), and bias/mean error (ME)—are the mean value of that metric over the 100 iterations of holdout validation. Variable importance, calculated as the percent increase in mean squared error if each variable in the model were randomized one at a time was also averaged over the 100 iterations. Because several predictor variables were highly correlated (*r* > 0.8) (Figure S1), the robustness of the variable importance results was evaluated by pruning 7 correlated predictor variables from the models in an additional 100-iteration Monte Carlo random forest regression analysis. Sensitivity in variable importance was also evaluated in a Monte Carlo 100-iteration random forest regression analysis using only PFOS concentration data in the fish tissue samples (PFOS detections in 31 samples) in order to evaluate the influence of the high range of PFOS concentrations in the ∑PFAS model results. The small number of fish tissue samples with PFAS measurements other than PFOS (*n* = 17, 38%) did not allow for a meaningful analysis of results for models using the other chemicals alone due to the heavy zero-inflation. Partial dependence plots were developed from a random forest regression model using the complete matched fish tissue dataset (*n* = 45). For ∑PFAS predictions at all of the waterbody points (*n* = 1039) in Washington and Oregon, the model was trained using all matched fish tissue occurrence data (*n* = 45) and then predicted onto all points. All random forest models were constructed using 1000 trees with an mtry (number of predictors considered at each decision tree split) of 10, which maximized the percent of variance explained by the model.

Random forest classification models were also developed and evaluated with the same Monte Carlo holdout validation scheme as the regression models described above. However, instead of a continuous response variable, the fish tissue ∑PFAS concentrations in the classification model were recoded into two groups for concentrations above and below selected ∑PFAS concentration threshold values. A sensitivity analysis was conducted using these random forest classification models for three different ∑PFAS threshold concentrations—1.5, 3, and 5 ng/g. The threshold concentrations were arbitrarily chosen due to there not being a currently established federal standard or statewide standard in Washington or Oregon for PFAS concentrations in fish tissue and due to the limited range of concentrations in the compiled occurrence dataset. Evaluation metrics for each of the three threshold value models were averaged over the 100 iterations and included area under the curve (AUC), accuracy, sensitivity, and specificity. Variable importance, calculated as the average of each predictor variable’s mean decrease in accuracy over the 100 iterations, is also presented for each cutoff ∑PFAS concentration in the sensitivity analysis. The random forest classification models were constructed using 1000 trees with an mtry of 10. All statistics and modeling in this study were conducted using R version 4.2.1.^38^

## 3 Results and Discussion

### 3.1 Data Summary

The number of fish tissue samples from Washington in the final dataset for this study was 23, while the number of fish tissue samples from Oregon was 22, for a total of 45 samples. These samples were collected in 23 unique locations in Washington with data from 14 species of fish and in 18 unique locations in Oregon with data from 10 species of fish. Locations in Oregon sampled at the same location but during different years were in the Willamette River, Tualatin River, Sandy River, and Rogue River. In comparison to the eastern U.S., the Columbia River Basin has relatively little fish tissue PFAS data that are publicly available.^23^

**Figure 2:**
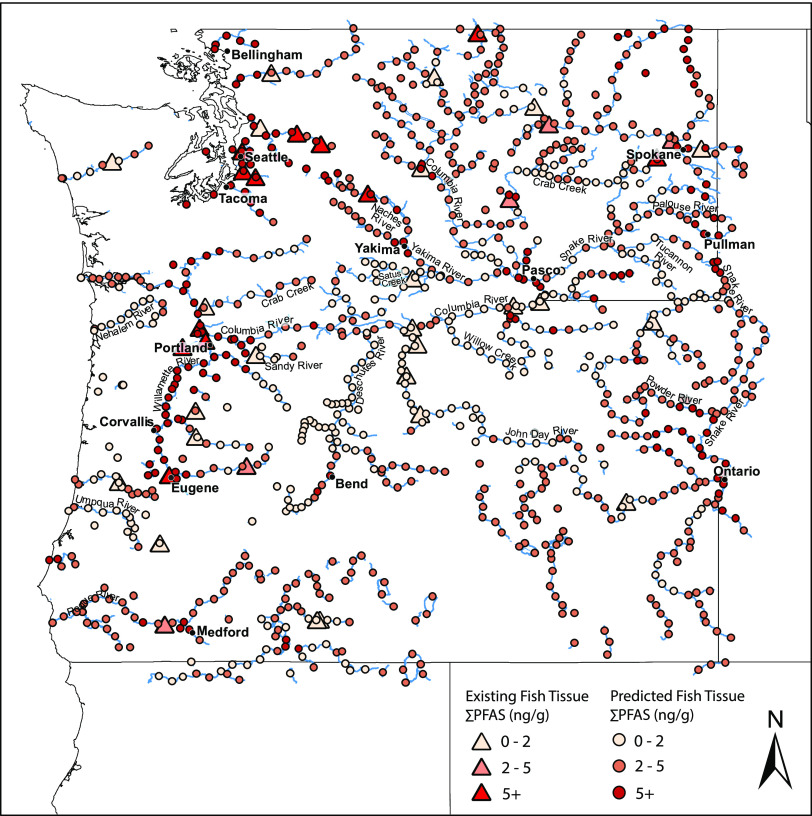
Map of Washington and Oregon showing predicted ∑PFAS concentrations (shaded circles) in fish tissue (fillet, skin on) from a random forest regression model in rivers and lakes selected based on previous fish tissue sampling data locations and the likelihood of fishing activities. Existing ∑PFAS measurement data in fish tissue (fillet, skin on) are shown as shaded triangles.

Of the 10 measured PFAS that were common between the fish tissue datasets, only 6 chemicals were detected in at least 1 sample—PFDA, PFDoA, PFHxS, PFNA, PFOS, and PFUnA. Summary statistics for all PFAS detected in this study’s fish tissue dataset are shown in Table 1. The most frequently detected (69%) PFAS in the fish tissue samples was PFOS, and the chemical with the highest mean (5.85 ng/g) and maximum (74.20 ng/g) concentrations was also PFOS. This dominance of PFOS in fish tissue has been observed in several other studies of PFAS in fish tissue throughout the U.S.^39−42^ PFUnA was detected next most frequently in 33% of the samples with a mean concentration of 0.33 ng/g and maximum concentration of 5.31 ng/g. Detection frequencies for PFDA, PFDoA, and PFNA were 20, 18, and 16%, respectively. Mean concentrations for PFDA, PFDoA, and PFNA were 0.34, 0.21, and 0.10 ng/g, respectively. PFHxS was only detected in one sample at 0.73 ng/g. When PFAS concentrations for all chemicals measured in each sample were summed, 73% of the fish tissue samples had a detection of ∑PFAS above zero (*n* = 33). The mean ∑PFAS concentration was 6.83 ng/g and maximum ∑PFAS concentration was 87.29 ng/g. Much of the existing fish tissue occurrence data used in this study comes from sampling conducted near larger cities in Washington and Oregon such as Seattle, Spokane, and Portland (Figure 2).

**Table 1:** Summary Statistics for PFAS Measurements in Fish Tissue (Fillet, Skin On) Samples from Washington and Oregon^a^

	N	N > LOD^b^	max	AM	SD	50th percentile	75th percentile	95th percentile
PFOS	45	31	74.20	5.85	12.72	0.89	6.40	28.18
PFUnA	45	15	5.31	0.33	0.87	0.00	0.33	0.91
PFDA	45	9	4.31	0.34	0.93	0.00	0.00	2.74
PFDoA	45	8	3.47	0.21	0.65	0.00	0.00	1.10
PFNA	45	7	0.87	0.10	0.25	0.00	0.00	0.74
PFHxS	44	1	0.73					
**∑PFAS**	**45**	**33**^c^	**87.29**	**6.83**	**14.83**	**1.60**	**6.56**	**33.96**

a Measurements below the limit of detection (LOD) were substituted with zero.

b Number of samples for which measurements were above the limit of detection.

c Number of samples for which at least one PFAS was measured above the limit of detection.

At the time of publishing this article, fish consumption advisories and recommendations in the U.S. are issued at the state or local levels, which has only been done for PFAS in fish in 14 states.^40^ Fish consumption advisories for PFAS have been issued primarily for PFOS, including an updated meal allowance recommendation for the Columbia Slough watershed in Oregon and three lakes in Washington state in 2022.^43,44^ The fish tissue dataset used in this study suggests that PFOS was the main potential contributor to consumer PFAS exposure from fish in the Columbia River Basin region. Nationally, the highest total PFAS concentrations were generally found outside of the Columbia River Basin.^40^ The median concentration for PFOS in fish tissue in Washington and Oregon in this study (0.89 ng/g) was less than the median found in nationwide fish tissue samples (6.6 ng/g).^40^ Median total PFAS concentration in the national study (9.51 ng/g) was higher than the median ∑PFAS concentration observed in this study (1.60 ng/g).^40^

### 3.2 PFAS Predictions for Fish Tissue from Random Forest Regression

A map showing ∑PFAS predictions in fish tissue throughout the selected waterbodies in Washington and Oregon from the random forest regression model is shown in Figure 2. Prediction concentrations ranged from 0.68 to 58.10 ng/g with a right skewed distribution similar to that seen in the existing fish tissue occurrence dataset. Predicted ∑PFAS concentrations in fish tissue that were less than 2 ng/g accounted for 31% of the waterbody points, while predicted ∑PFAS concentrations in fish tissue greater than 5 ng/g accounted for 18% of the waterbody points. The highest predicted concentrations, where ∑PFAS was greater than 10 ng/g, accounted for 8% of the waterbody points. Areas with ∑PFAS predictions greater than 5 ng/g are thought to be mainly driven by PFOS contamination, while some areas with lower ∑PFAS concentrations might be driven by other PFAS.

The highest ∑PFAS concentrations in fish tissue were generally predicted to occur near cities with larger populations such as Seattle, WA, Tacoma, WA, Spokane, WA, Portland, OR and Eugene, OR. These more populated areas with higher densities of potential PFAS sources tend to also be areas targeted for PFAS sampling in various media, which was evident in the fish tissue occurrence dataset. However, the predictions in this study also suggest potential for higher ∑PFAS concentrations (>10 ng/g) in fish tissue in previously unsampled areas such as those near Clarkston, WA, Pasco, WA, Pullman, WA, Metaline Falls, WA, Bellingham, WA, Bend, OR, Hereford, OR, and Ontario, OR (Figure 2). The authors are not aware of any publicly available environmental media measurements of PFAS to provide evidence for potentially high concentrations in these areas, highlighting the need for future investigations and sampling in this region.^23^ Other unsampled areas with the potential for intermediate levels (2–10 n/g) of PFAS contamination in fish tissue include those along the Columbia River and its tributaries in northeast Washington, the Yakima and Naches Rivers in southcentral Washington, the Palouse River in southeastern Washington, the Snake and Powder Rivers in northeast Oregon, and the Rogue River in southwest Oregon (Figure 2). Areas in which lower ∑PFAS concentrations are predicted to occur in fish tissue include the Tucannon River in southeast Washington, Crab Creek in east central Washington, Satus Creek in south central Washington, the Deschutes and John Day Rivers and Willow Creek in north central Oregon, and the Nehalem and Umpqua Rivers in western Oregon.

**Figure 3:**
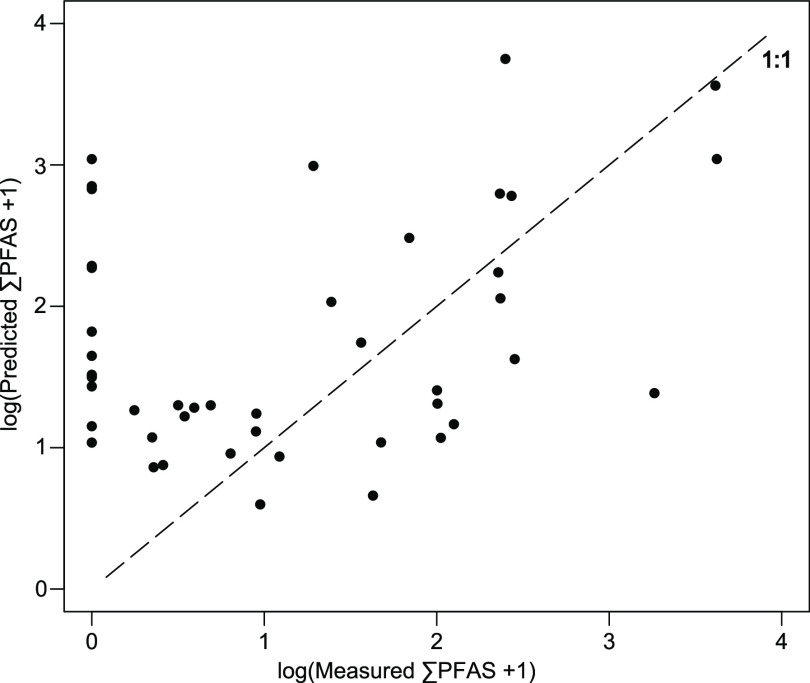
Out-of-bag modeled ∑PFAS predictions (log + 1) from a random forest model plotted against measured ∑PFAS values (log + 1) in fish tissue (fillet, skin on). Dashed line shows 1 to 1 relationship.

The random forest regression model was evaluated using a 100-iteration Monte Carlo scheme where the data were randomly split into training data and holdout data 100 times over which the error metrics were averaged. The mean MAE was 7.26 ng/g, which is 8.32% of the range of measured ∑PFAS occurrence in the fish tissue samples, while the mean RMSE was 164.65 ng/g. Additional training data in the higher ranges of ∑PFAS concentrations, as well as lower instrumentation limits of detection to lessen the number of samples with nondetects, could improve future model performance. The mean ME, or bias, over the 100 iterations was 1.14 ng/g, indicating that there is a small bias toward overpredicting ∑PFAS concentrations in the samples. Out-of-bag model predictions are plotted against measured values from a single random forest model to visualize model performance over the entire range of measured ∑PFAS concentrations in the dataset (Figure 3). The model generally performed poorly for samples with nondetect (zero) or low ∑PFAS concentrations, which were overpredicted by the model. Mid and high ranges of ∑PFAS concentrations were both over- and underpredicted similarly.

**Figure 4:**
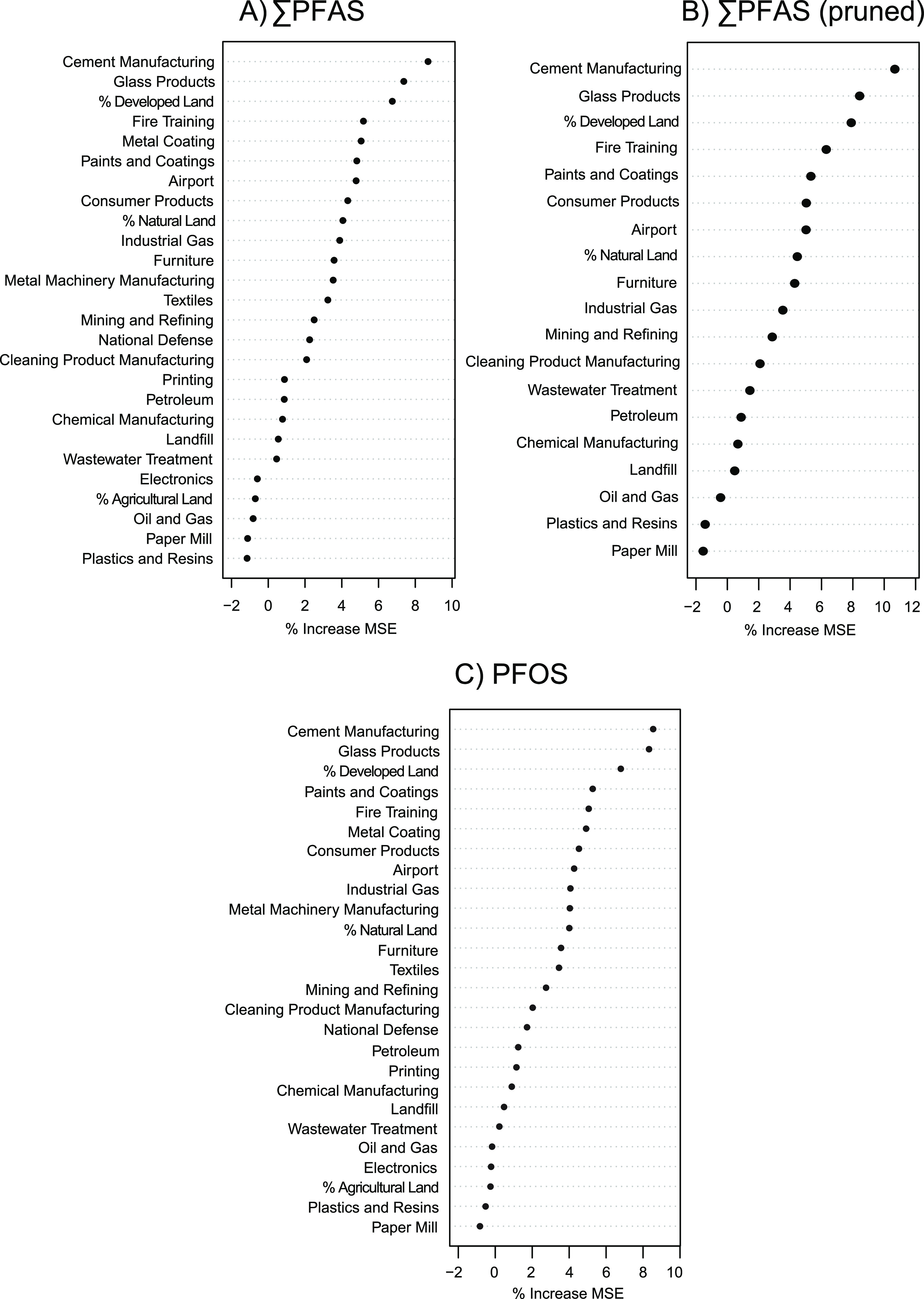
Mean variable importance from 100-iteration Monte Carlo random forest regression models for (A) ∑PFAS, (B) ∑PFAS pruned by removing highly correlated predictors, and (C) PFOS predictions in fish tissue (fillet, skin on). Metric used to determine variable importance is the percent increase in mean square error (MSE).

Over the 100 iterations, the variables in the random forest regression models with the highest mean percent increase in MSE, thereby implying that those variables were important drivers of ∑PFAS predictions, were the distance from the nearest cement manufacturing facility followed by the distance from the nearest glass product facilities (Figure 4). However, cement manufacturing and glass product facilities are some of the least represented industries in these states (9 and 15 facilities, respectively) (Figure S3).^23^ Other variables that were important predictors in the regression model were the percent of developed land, distance from the nearest fire training facility, distance from the nearest metal coating facility, distance from the nearest paints and coatings facility, and distance from the nearest airport. Variables that were not important in driving ∑PFAS predictions, where the model performed better without their inclusion, were the distance from the nearest electronics facility, percent of agricultural land, distance from the nearest oil and gas facility, distance from the nearest paper mill facility, and distance from the nearest plastics and resins facility. These industries represent some of the more well-represented industries (*n* > 65) in the region except for oil and gas, for which there is only one facility in central Washington. Boxplots showing the variability in the percent increase in MSE for each variable over the 100 iterations is shown in Figure S2.

In order to assess the potential influence on the variable importance results from highly correlated predictor variables (*r* > 0.8, Figure S1), 7 predictors (textiles, printing, metal machinery manufacturing, metal coating, electronics, national defense, and percent agricultural land) were removed for a pruned 100-iteration Monte Carlo random forest regression analysis. When these highly correlated variables were removed, the top 3 variables of importance in the models (cement manufacturing, glass products, and percent developed land) remained unchanged, and similar variables are shown as important and unimportant between the pruned and unpruned analyses (Figure 4). This indicates that the variable importance results were robust even with highly correlated predictors included in the model. To assess the influence on variable importance results from possible PFAS species-specific sources, another Monte Carlo 100-iteration random forest regression analysis using only PFOS concentration data was performed. The PFOS-only models showed similar variables of high and low importance to that of the ∑PFAS models, indicating that the large range of concentrations of PFOS in the fish tissue is likely the main driver of variable importance results for the ∑PFAS models (Figure 4). With additional data collection and increased detections of other PFAS chemicals in fish tissue, a similar model could be developed in the future to investigate drivers and sources of non-PFOS PFAS in fish.

The distance to expected sources of PFAS contamination like airports and fire training facilities, often associated with elevated levels of PFOS from suspected or known AFFF use, were important predictors of PFAS contamination in environmental media in both this study and previous studies.^6,9,41,45^ Urban land use has also been shown to be an important predictor of PFAS contamination in this and a groundwater study.^6^ However the distance to cement manufacturing and glass product facilities were not expected to be important predictors of PFAS contamination. A previous study predicting PFAS in groundwater did not find either industry to be important variables in their model.^6^ Uses of PFAS in cement manufacturing include being added to reduce cement shrinkage, maintain the cement’s flowing ability without increasing water content, and protect the cement from natural elements and pollutants.^1,46^ Cement has also been used for PFAS remediation where contaminated soils and sediments are incorporated into cements and concretes as fine particle aggregates.^47^ Glass product industries also use PFAS to protect the glass from weather and pollutants.^46^ In addition, PFAS are used on glass as an anti-mist coating to prevent fogging on mirrors, automobile windshields, eyeglasses, and greenhouses.^46^ Glass etching facilities also use PFAS as a wetting agent.^46^

These differences in important model predictors between environmental media types could suggest that some media are contaminated by PFAS differently than other media, whether it be due to PFAS structure and chemical properties, the distance PFAS can travel from a source in various media types, bioaccumulation in living media, or the influence of air deposition in media near the surface versus groundwater. Differences between the influence of certain industries and PFAS contamination in environmental media in different studies could also be due to regional differences in hydrogeology, biota, and fauna, and the prevalence of different industries. However, the inflated influence of PFOS in environmental media models could cause important drivers of other PFAS chemicals from smaller sources to be overlooked, which should be considered during future study design and interpretation of results.

**Figure 5:**
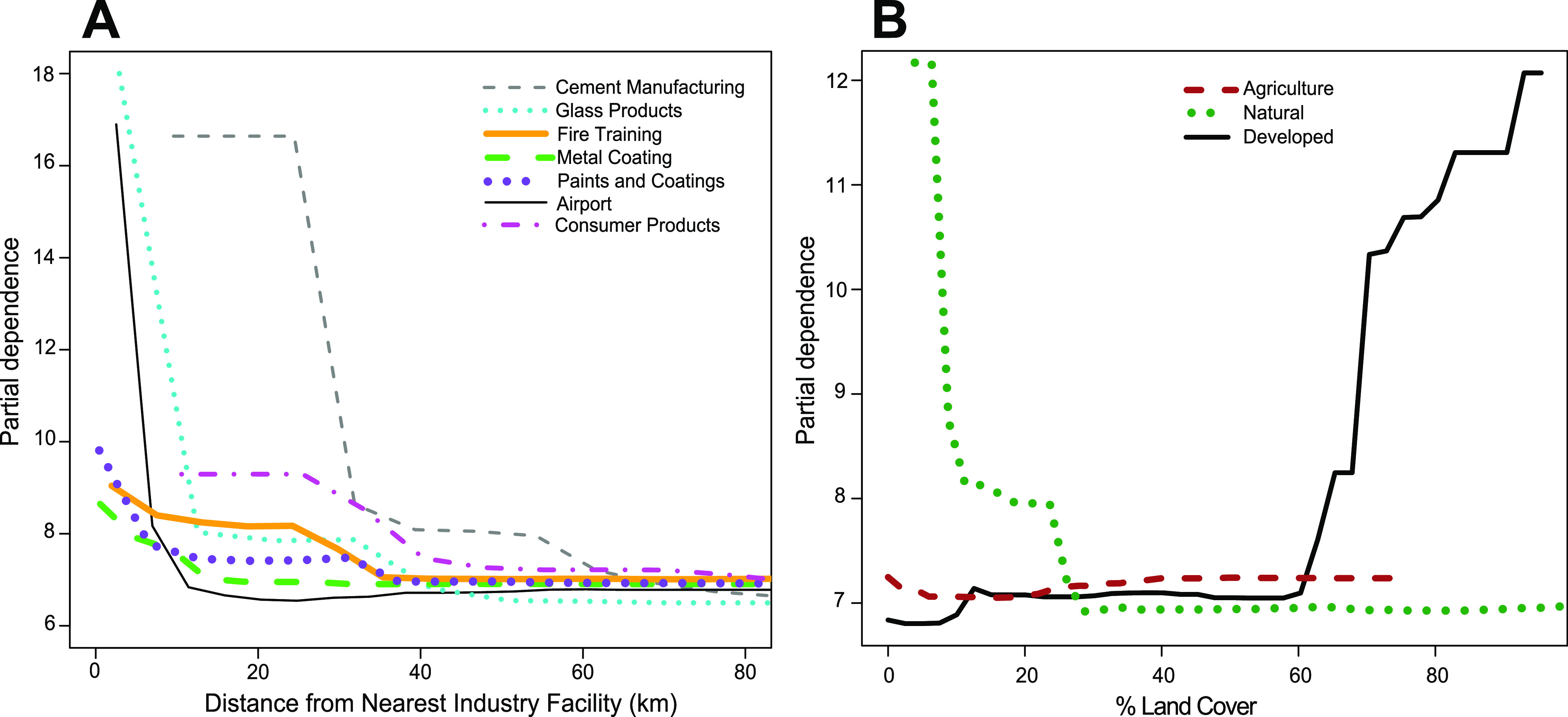
Partial dependence plots from the random forest regression model showing marginal effects on ∑PFAS concentrations in fish tissue (fillet, skin on) for (A) distance from the nearest industry facilities (for top 7 industries from variable importance plot) and (B) percent land cover.

Partial dependence plots for the top industries in the variable importance analysis show a relationship between the distance from the nearest industry facility and ∑PFAS concentrations in fish tissue, giving insights into how large of a radius from a PFAS-related facility one might find contaminated fish. In this study, ∑PFAS concentrations were elevated in fish tissue up to about 35 km from cement manufacturing facilities, with smaller elevations in ∑PFAS seen in fish up to about 60 km from cement manufacturing facilities (Figure 5). Elevations in ∑PFAS were found in fish tissue located about 17 km from glass product facilities, with smaller elevations observed up to about 40 km from those sources. Elevations in ∑PFAS were not observed past about 16 km from airports. Smaller elevations in fish tissue ∑PFAS were observed up to about 35 km from fire training sites, 7 km from paints and coatings facilities, 40 km from consumer products facilities, and 15 km from metal coating facilities. The influence of land cover on fish tissue ∑PFAS concentrations was also analyzed using partial dependence plots (Figure 5). Elevations in ∑PFAS were observed when the percentage of natural land surrounding a location was below about 28% and the percentage of developed land was above about 60%. While there were comparatively few locations surrounded by agricultural land in this study, a small increase in ∑PFAS in fish tissue was observed when the percent of agricultural land was above about 20%. While fate and transport models for surface water flow or airflow could better estimate how far PFAS contamination may travel from these industry facilities, semi-quantitative tools like partial dependence plots can help to estimate the fate and transport of PFAS in other complex media such as fish in a data-driven approach.

### 3.3 PFAS Predictions for Fish Tissue from Random Forest Classification

Maps showing ∑PFAS predictions in fish tissue from random forest classification models throughout selected waterbodies in Washington and Oregon are shown in Figures S4–S6. Three cutoff concentrations were used to classify ∑PFAS predictions as either detects or nondetects in fish tissue—1.5, 3, and 5 ng/g. Due to their not being current statewide (in Washington state or Oregon) or federal health advisories for PFAS concentrations in fish, these cutoff concentration values were chosen arbitrarily based on the limited range and distribution of concentrations in this dataset to illustrate the random forest classification method, show sensitivity in results with varying thresholds, and compare classification results to those from the regression model. Regulators could find this classification methodology useful by choosing a threshold concentration value meaningful to their particular health advisory, chemical, or potentially exposed populations that would allow them to identify hotspots of PFAS in fish tissue in their regions of interest.

Concentrations in fish tissue above those thresholds (1.5, 3, and 5 ng/g) were predicted in fish tissue at 32, 12, and 7% of the waterbody points, respectively. Similar to the regression model predictions, the classification models predicted detections above the thresholds mainly near more populated cities like Seattle, WA, Spokane, WA, Tacoma, WA, Eugene, OR, and Portland, OR. For the 5 ng/g threshold model, only a handful of waterbody points farther away from these larger cities, primarily in northcentral Washington state, were predicted to have fish tissue ∑PFAS concentrations above the threshold. In contrast, fish tissue concentrations were more likely to be predicted to be above the threshold in the 1.5 ng/g classification model farther away from major cities along the same waterbodies as well as along waterbodies that do not intersect with these larger cities such as those in northeastern Washington and southcentral Oregon. More populated areas with higher densities of potential PFAS sources, particularly those with known or suspected AFFF use, have historically been areas that were targeted for PFAS sampling. Here, most of the areas with predicted detections in the highest concentration cutoff value model, 5 n/g, are those which have already been sampled and are likely driven by the higher range of PFOS concentrations in fish tissue. However, lowering the threshold concentration in the classification models could help identify additional, previously unsampled areas or areas driven by contamination other than AFFF use in which future efforts could be focused.

The random forest classification models, using three cutoff ∑PFAS concentrations for detects or nondetects in the fish tissue, were evaluated with a 100-iteration Monte Carlo analysis. The best-performing classification model was the 5 ng/g threshold concentration, where the mean AUC, accuracy, sensitivity, and specificity over the 100 Monte Carlo iterations were 0.79, 81.78%, 80.63%, and 85.08%, respectively. The worst-performing classification model was the 1.5 ng/g threshold concentration, where the mean AUC, accuracy, sensitivity, and specificity over the 100 Monte Carlo iterations were 0.63, 71.00%, 65.12%, and 79.42%, respectively. For the 3 ng/g threshold model, the mean AUC, accuracy, sensitivity, and specificity from the classification model over the 100 Monte Carlo iterations were 0.72, 80.22%, 74.62%, and 84.42%, respectively. This difference in performance between the three thresholds indicates that the classification models were able to better distinguish between higher concentrations and nondetects than lower concentrations and nondetects in the fish tissue. This may be due to indirect sources driving lower PFAS concentrations in fish tissue, while higher PFAS concentrations are more likely driven by sources with direct PFOS contamination. In all three models, the specificity was higher than the sensitivity, meaning that the models were able to correctly identify nondetects better than they were able to correctly identify detections above the thresholds.

For the better performing classification models (mean AUC > 0.7) with cutoff ∑PFAS concentrations at 3 and 5 ng/g, the variable with the highest mean decrease in accuracy, therefore implying that it was an important driver of ∑PFAS detects or nondetects at those thresholds, over the 100 iterations was distance from the nearest paints and coatings facility (Figure S7). For the 3 ng/g threshold model, other important variables in the models were distance from the nearest metal machinery manufacturing, distance from the nearest landfill, distance from the nearest metal coating facility, distance from the nearest mining and refining site, distance from nearest fire training site, and percent developed land. For the 5 ng/g threshold model, other important variables in the models were the nearest metal coating facility, distance from the nearest metal machinery manufacturing, distance from the nearest industrial gas facility, percent developed land, distance from the nearest glass products facility, and distance from nearest wastewater treatment plant. While the 1.5 ng/g classification model did not perform as well as the previous models (mean AUC = 0.63), its top variables of importance were the distance from the nearest fire training site, percent developed land, percent natural land, distance from the nearest mining and refining site, distance from the nearest landfill, and distance from the nearest airport (Figure S7).

The distance from the nearest paints and coatings facility was the highest variable of importance in the 3 and 5 ng/g threshold classification models and was also a higher variable of importance in the regression models (Figures 4 and S7). A previous study did not find the distance from paints and coating facilities to be an important variable for predicting PFAS in groundwater.^6^ There are 65 paints and coatings facilities listed in Washington and Oregon, but most are located in developed areas and near larger cities (Figure S3).^23^ Household paints have used PFAS as fluorosurfactants for leveling, surface wetting, gloss, oil and water repellants, and as anti-blocking agents on interior doors and walls.^1,48^ PFAS are also used in paints for chemical reaction vessel linings, increasing weatherability and durability of bridges, and aerosol spray paints used on cars.^48^ Coatings containing PFAS have been used for high performance wiring and cables, exterior surfaces of buildings and bridges, electronics screens, and semi-conductors.^48^ PFAS are used in coatings for anti-stick, anti-corrosive, anti-reflective, and fire-resistant properties.^1,48^ One study found that PFOS was the main PFAS detected in wet room sealing paint,^49^ which could help explain the high variable importance of distance to the nearest paints and coatings facility for the higher threshold concentration classification models (3 and 5 ng/g) and the regression models due to the larger range of PFOS concentrations in the dataset compared to the other chemicals. Other similar variables of higher importance between the classification and regression models were the distance from the nearest metal coating facility and percent developed land (Figure 4). The metal coating industry has many facilities in Washington and Oregon (*n* = 290), but similar to the paints and coatings industry, there are few metal coating facilities located outside of developed areas and larger cities (Figure S3).^23^

Notably, the top variable of importance in the regression models, the distance from the nearest cement manufacturing facility, was not found to be a top variable of importance for the classification models. While it was near last of importance in the 1.5 ng/g classification model, it moved up in importance in the 3 and 5 ng/g classification model, with it being the highest in the list of important variables in the 5 ng/g model. The effects of this disagreement between models can be noted in northeastern Washington state, where a cement manufacturing facility (Figure S3) appears to drive higher ∑PFAS predictions in the regression model (Figure 2) that are not as apparent in the classification model predictions (Figures S4–S6). While glass products were important in both the regression models and the highest threshold concentration classification model (5 ng/g), it was not an important predictor in the lower threshold concentration classification models. This may suggest that the distance from the nearest cement manufacturing facility and the distance from the nearest glass products facility is important in detecting higher ranges of ∑PFAS concentrations in fish tissue, particularly PFOS concentrations, while they are less important when detecting lower ∑PFAS concentrations.

### 3.4 Strengths and Limitations

Random forest models like those used in this study tend to outperform other predictive models because their averaging structure minimizes over-fitting issues, which cause many other machine learning algorithms to lose generalizability.^37^ These models are useful for nonparametric and high-dimensional data because they do not require data transformations and model performance is relatively insensitive to multicollinearity. Random forests can also give insights into potentially complex, nonlinear, or unknown relationships in the data that influence the modeling output using variable importance plots and partial dependence plots.^50^ Based on the application, random forests can be used to model and predict either continuous or categorical data. As demonstrated here, classification models may be useful for applications in which a set threshold concentration for PFAS in environmental media has been determined. While the thresholds used here were arbitrary, regulators could use this methodology with any threshold concentration value that allows them to identify hotspots meaningful to their particular health advisory, chemical, or potentially exposed populations.

However, a limitation of random forests is that they are not able to predict concentrations outside of the range of the training dataset. Predicted ∑PFAS concentrations near the higher range of the training dataset used in this study could potentially be much higher than the predicted value when sampled in situ. Therefore, until more data are available to train more robust models, these predictions are intended to be used to identify areas with the potential for higher or lower ∑PFAS concentrations in fish tissue that could be prioritized for future sampling and not as definitive quantitative concentration predictions.

Additionally, variable importance measures from random forest models can be sensitive to multicollinearity and varying magnitudes of predictors.^51,52^ While the variable importance results for the random forest regression models in this study did not appear to be sensitive to the removal of several highly correlated predictors, this limitation should be considered when interpreting results and investigating potential sources of PFAS contamination in the region. Because many of the industries identified as important in the random forest models are clustered near larger cities and industrial centers, it is difficult to tease apart definitive relationships between high levels of PFAS contamination from these industries due to their own emissions versus their frequent proximity to other emitters of PFAS. Conversely, industries appearing to be unimportant for predicting high levels of PFAS in the models may have a higher proportion of facility sites located away from urban industrial areas so that many of those facilities are not as frequently co-located with other emitters. Therefore, variable importance results from this study should be used to develop hypotheses for future study design and sampling strategies to further investigate the potential importance of these sources for environmental PFAS contamination.

This study, as well as several other PFAS modeling studies, uses ∑PFAS as the modeling output in the environmental media.^6,40,41^ While many previous studies have focused on modeling large concentration ranges of PFOS and PFOA contamination from AFFF, including other PFAS species with lower concentrations in the predictive models could give insights into smaller, less studied, or less reported sources. However, a limitation in using ∑PFAS concentrations which are dominated by PFOS concentrations is that these insights from other PFAS chemicals might be missed. Due to the small sample size of fish tissue samples available for this study, there was limited information to link specific sources to different PFAS. With more data and higher percentages of detections of multiple PFAS in fish tissue, modeling PFAS species separately could also help improve overall model performance and identify chemical-specific sources of contamination.

Future fish tissue modeling efforts in this region would also greatly benefit from additional fish tissue data from diverse locations. While previous PFAS sampling investigations have largely focused on areas with obvious high-level PFAS contamination from AFFF use around airports, fire training sites, and military installations, data points near other possible industrial sources would improve understanding of important drivers of PFAS contamination in fish and help to target of future remediation efforts. The propensity for previous sampling efforts being near larger and more populated cities also highlights a need for additional sampling in rural communities where other lesser-known or indirect sources may contribute to environmental contamination affecting the general population and populations more vulnerable to exposure because of environmental injustice concerns.

Particularly for fish in the Columbia River Basin, some of which are migratory species, sampling in additional locations would also help increase understanding of the fate and transport of PFAS from various sources in a less-studied and complex environmental media. Where fate and transport models can give insights into how far PFAS can travel from a source in some media like surface water and air, fish tissue models assessing the spatial extent of PFAS contamination may be less dependent on factors like water flow direction and elevation and therefore call for a more data-driven approach. Fish migration in the Columbia River Basin is also impacted by numerous hydroelectric dams along the major rivers which could impact modeling results.^53^ As the amount of available fish tissue data increases, modeling studies such as this could also begin to consider variability in PFAS concentrations in fish based on the species-specific physiology and trophic level, which has been observed previously.^39−41^ Future work could also account for differences in the accumulation of PFAS within various parts of fish such as the fillet, skin, and organs, the latter of which has been observed to contain higher concentrations.^54,55^

While more PFAS data currently exists for surface water and groundwater than fish tissue, studies have shown that the PFAS compositions in fish do not necessarily reflect that of the surrounding water, suggesting that prediction models should be developed for fish independently from water media.^39^ In addition to exposure from the surrounding surface water, PFAS exposure pathways for fish also include sources like sediment and their diet. This highlights an additional data need for paired fish tissue and environmental media samples in order to better understand drivers of fish’s PFAS exposure. The partitioning and elimination rates of PFAS in fish are also active areas of research that can inform future models, sampling, and health advisories.^41,56−59^

The scarcity of PFAS measurements in fish tissue available in the Columbia River Basin hindered the robustness and generalizability of the results from this work but also highlighted a continued need for data generation and modeling in the region. Hypotheses generated from this work and the demonstration of a generalizable, efficient methodology will help with the facilitation and design of future studies, sampling campaigns, and investigations of potentially important PFAS sources in the Columbia River Basin and beyond.

## Supporting Information Available

Spatial predictor correlations; variable importance boxplots over 100 Monte Carlo iterations; map of important industry locations; maps of classification model predictions; sensitivity analysis results from classification model variable importance (PDF)
