# Supplementary material for: Using Geospatial Data and Random Forest To Predict PFAS Contamination in Fish Tissue in the Columbia River Basin, United States
Source: Environ Sci Technol. 2023 Sep 5;57(37):14024–35. doi: 10.1021/acs.est.3c03670 (PMC10515492; doi:10.1021/acs.est.3c03670)
Supplement: Supplementary file 1 — es3c03670_si_001.pdf [file es3c03670_si_001.pdf]

SUPPORTING INFORMATION

Using geospatial data and random forest to predict PFAS contamination in fish tissue in the Columbia River Basin, United States

Nicole M. DeLuca[1*], Ashley Mullikin[1], Peter Brumm[2], Ana G. Rappold[1], Elaine Cohen Hubal[1]

[1]Center for Public Health and Environmental Assessment, Office of Research and Development, U.S. Environmental Protection Agency, Research Triangle Park, North Carolina, 27709, USA

[2]Region 08, Water Division, U.S. Environmental Protection Agency, Helena, Montana, 59626, USA

*Corresponding author.
E-mail address: deluca.nikki@epa.gov (N.M. DeLuca)

8 Pages, 7 Figures

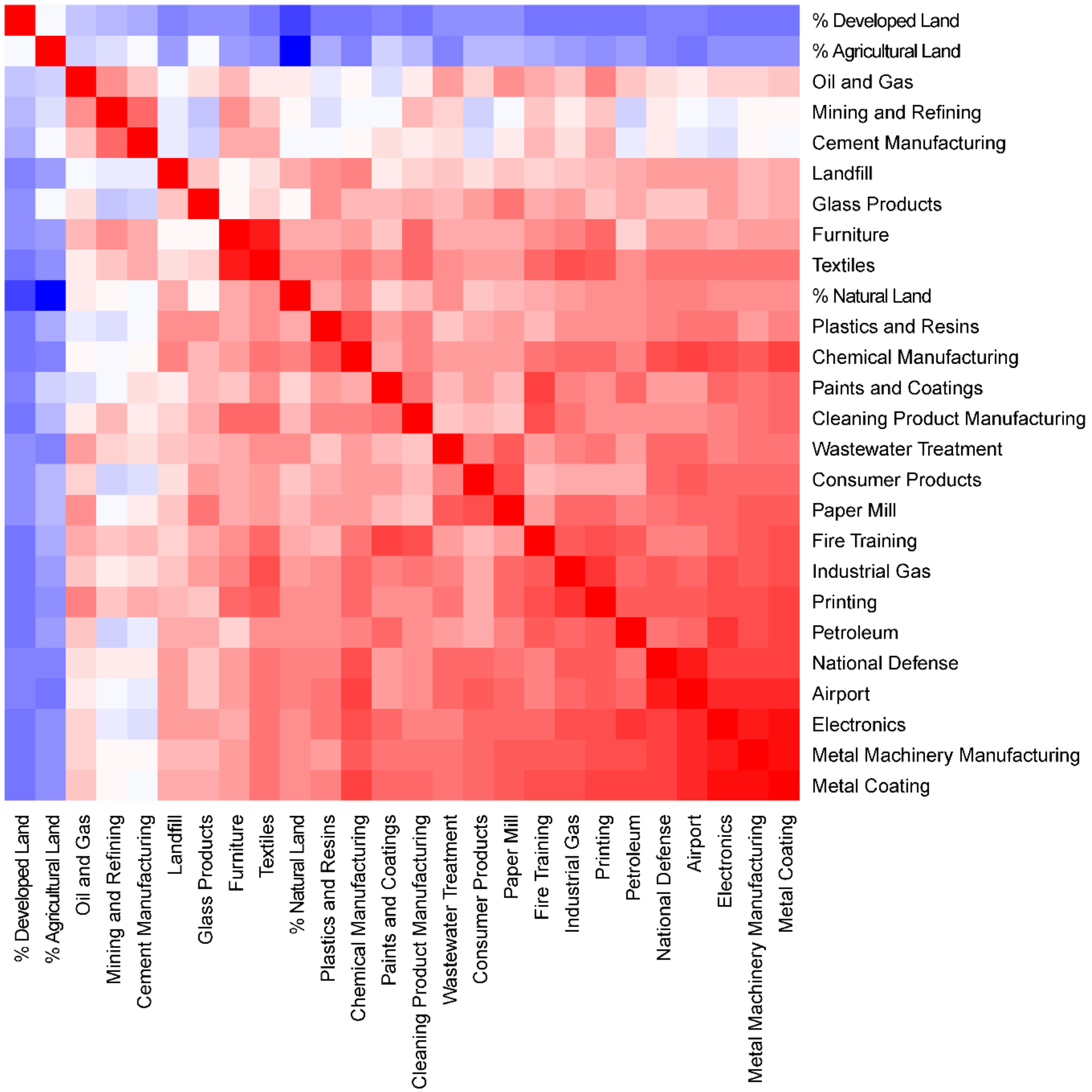

Figure S1. Pearson correlations between the quantified PFAS-related industry variables (distance to nearest). Darker red indicates correlation coefficient closer to 1, darker blue indicates correlation coefficent closer to -1, while white indicates a correlation coefficient of 0.

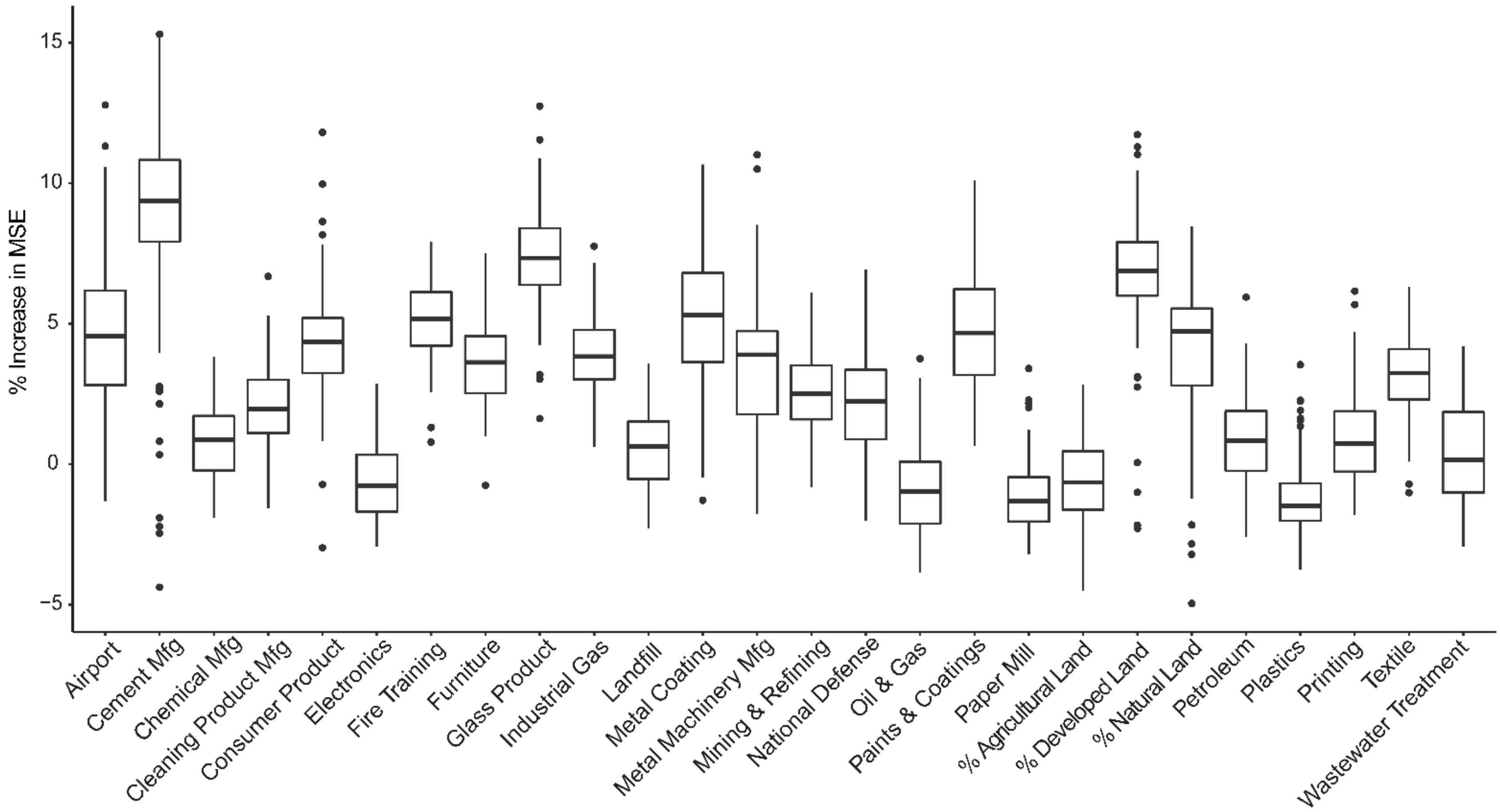

Figure S2. Boxplots showing variability in the percent increase in mean square error (MSE) for each predictor variable in the random forest regression models prediction ∑PFAS in fish tissue (fillet, skin on) over the 100 Monte Carlo iterations.

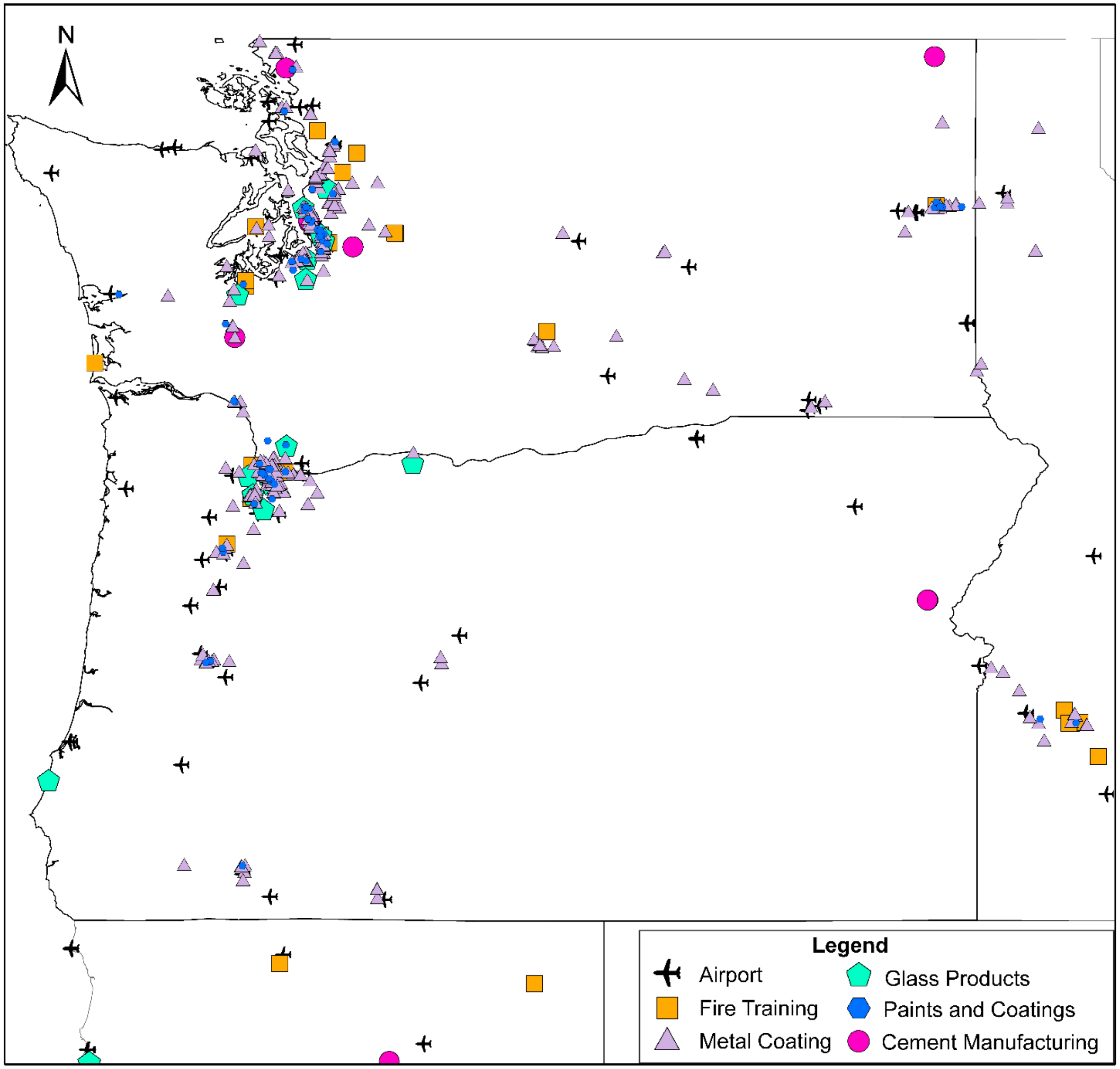

Figure S3. Map of Washington and Oregon showing important industries – airports (airplane symbol), fire training sites (orange squares), metal coating facilities (purple triangles), glass products facilities (teal pentagons), paints and coatings facilities (blue hexagons), and cement manufacturing facilities (pink circles) – from the random forest models' variable importance results.

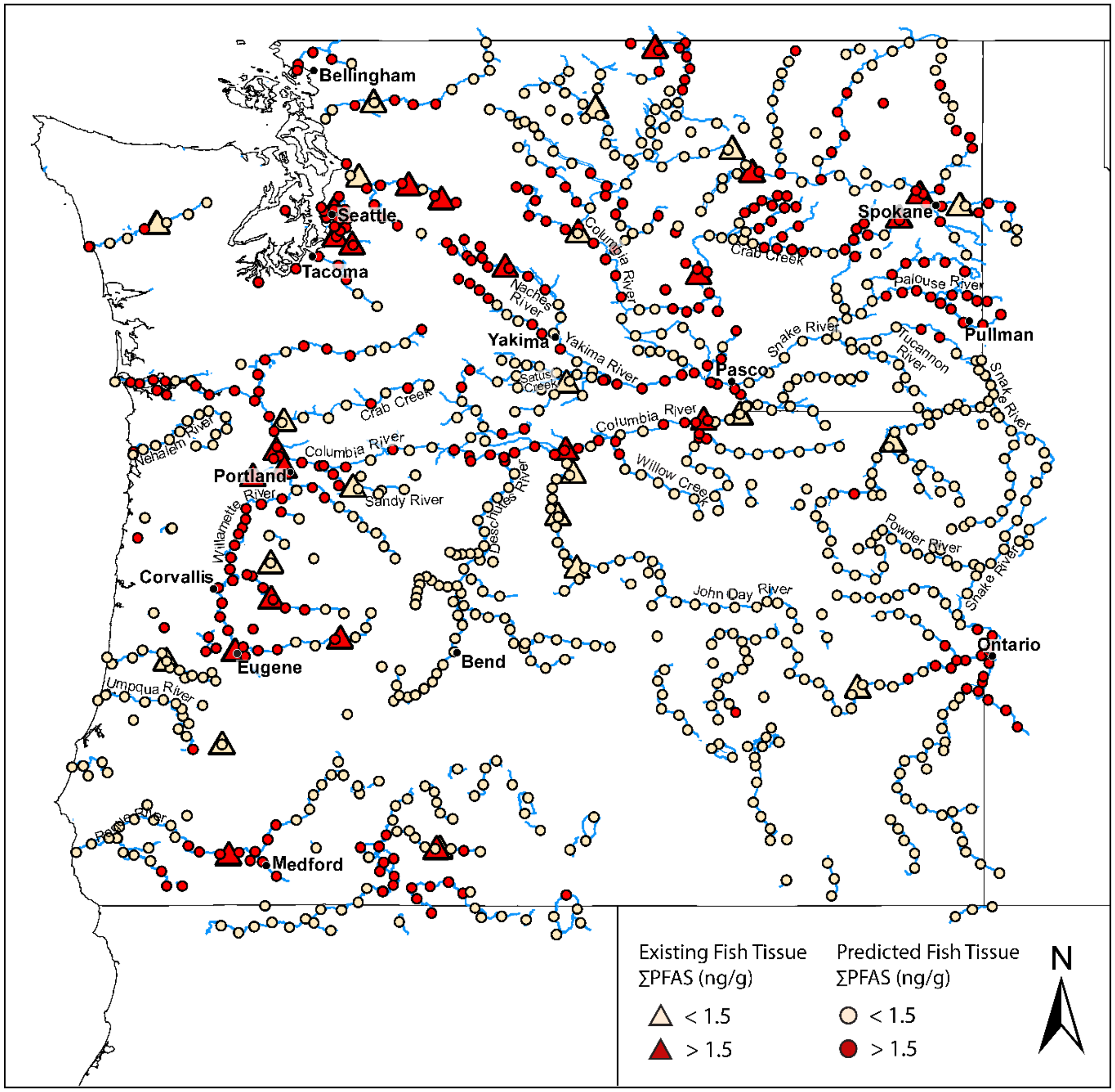

Figure S4. Map showing predictions in Washington and Oregon from a random forest classification model in which ∑PFAS concentration predictions (circles) in fish tissue (fillet, skin on) were classified into two groups – below 1.5 ng/g (beige) or above 1.5 ng/g (red). Existing fish tissue ∑PFAS data are shown as triangles and shaded the same as predictions.

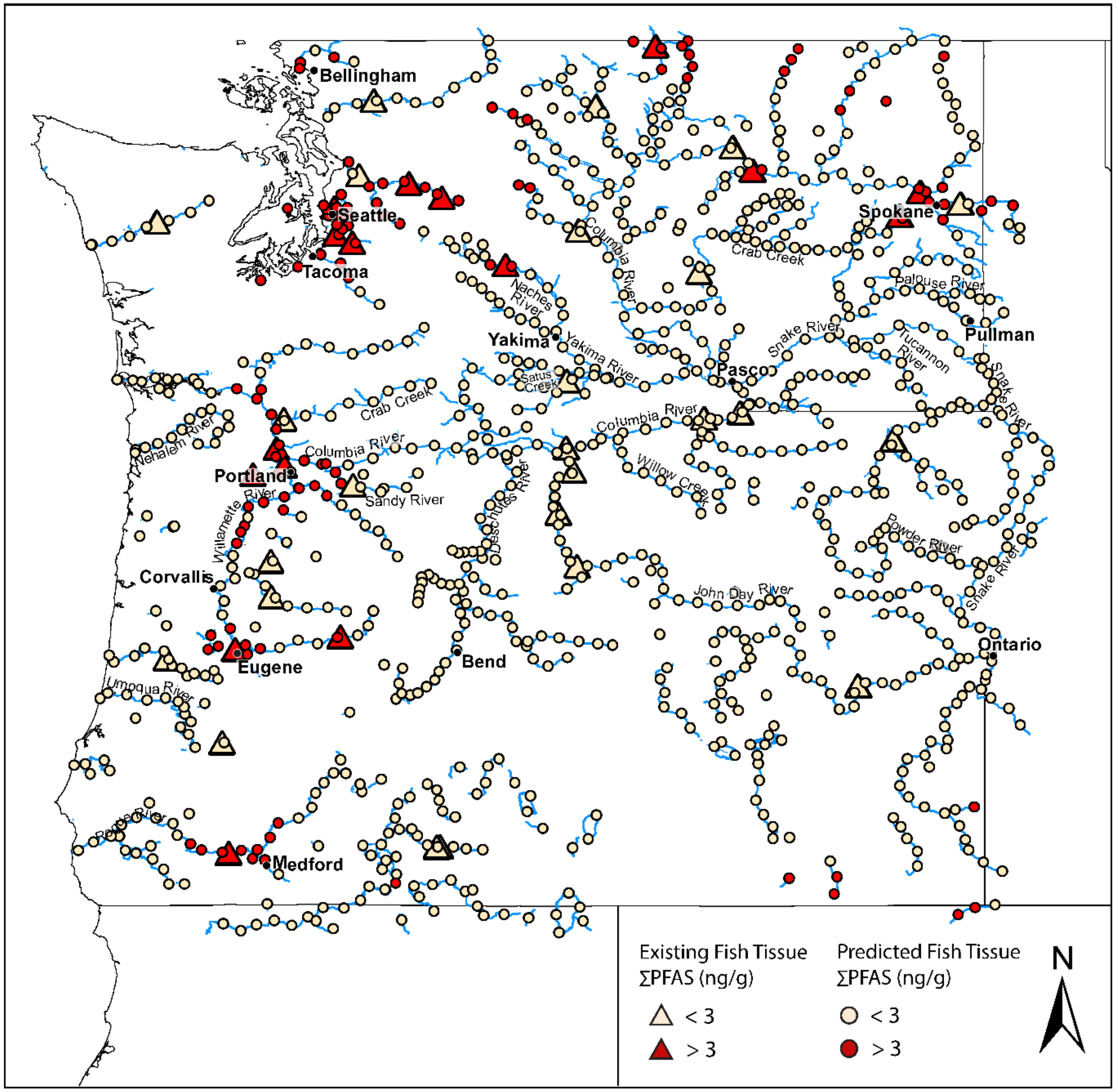

Figure S5. Map showing predictions in Washington and Oregon from a random forest classification model in which ∑PFAS concentration predictions (circles) in fish tissue (fillet, skin on) were classified into two groups – below 3 ng/g (beige) or above 3 ng/g (red). Existing fish tissue ∑PFAS data are shown as triangles and shaded the same as predictions.

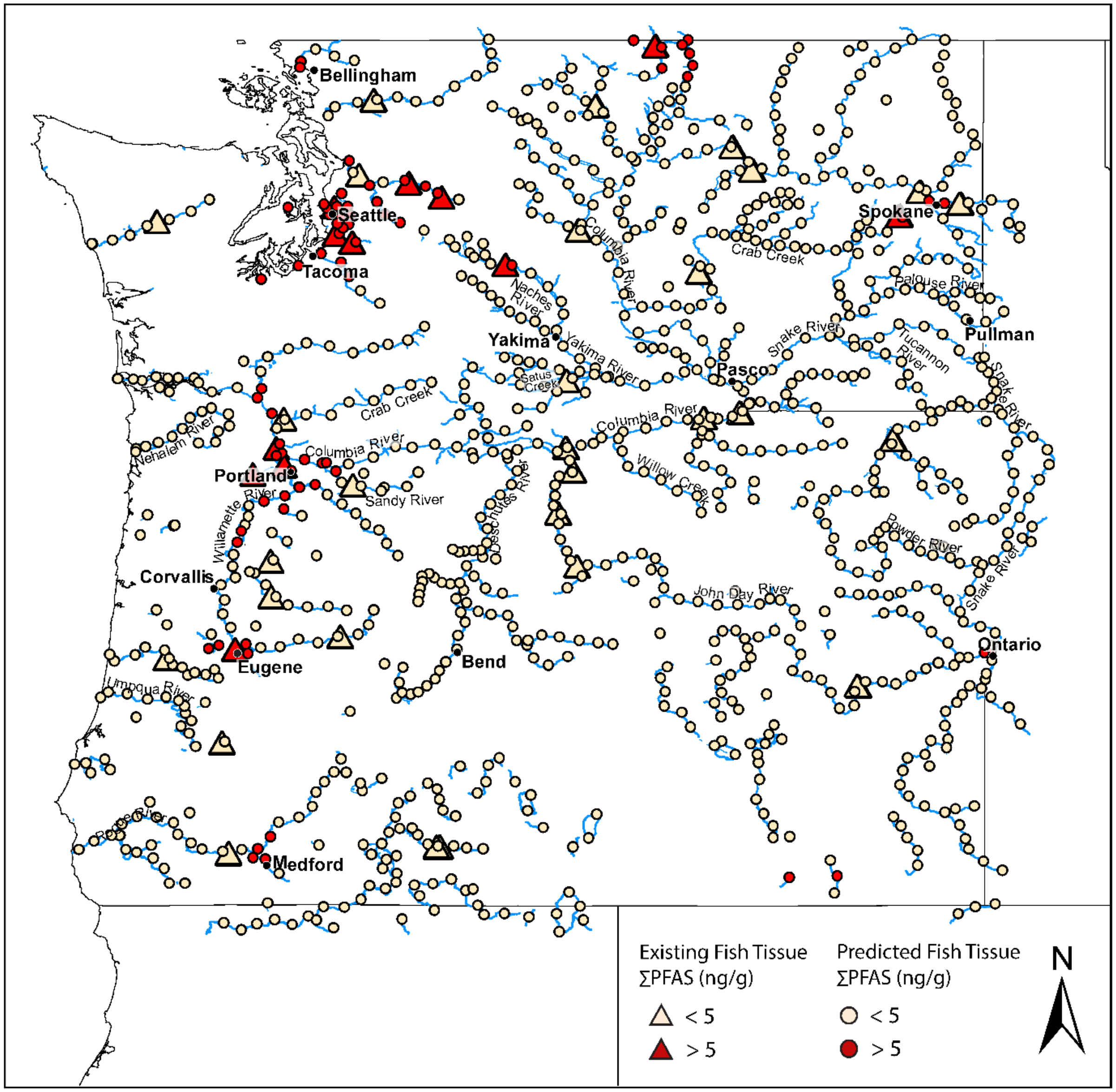

Figure S6. Map showing predictions in Washington and Oregon from a random forest classification model in which ∑PFAS concentration predictions (circles) in fish tissue (fillet, skin on) were classified into two groups – below 5 ng/g (beige) or above 5 ng/g (red). Existing fish tissue ∑PFAS data are shown as triangles and shaded the same as predictions.

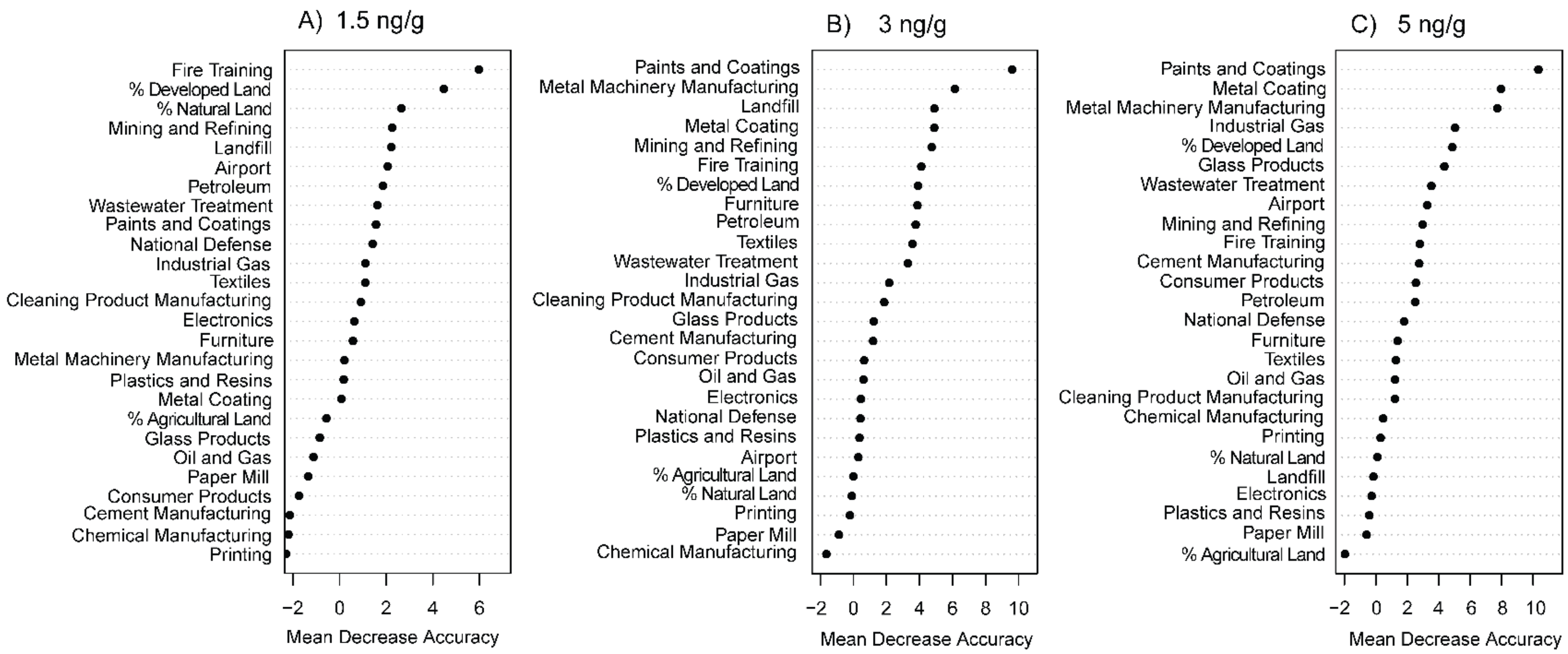

Figure S7. Variable importance sensitivity analysis results using random forest classification models with cutoff values between predicted ∑PFAS detects and non-detects at A) 1.5 ng/g, B) 3 ng/g, and C) 5 ng/g in fish tissue (fillet, skin on).
